# Evaluating the Extraction and Quantification of Marine Surfactants from Seawater through Solid Phase Extraction and Subsequent Colorimetric Analyses

**DOI:** 10.1021/acsestwater.4c00497

**Published:** 2024-10-25

**Authors:** Rachel L. Bramblett, Amanda A. Frossard

**Affiliations:** Department of Chemistry, University of Georgia, Athens, Georgia 30606, United States

**Keywords:** surfactants, seawater, solid phase extraction, colorimetry, UV–vis spectroscopy

## Abstract

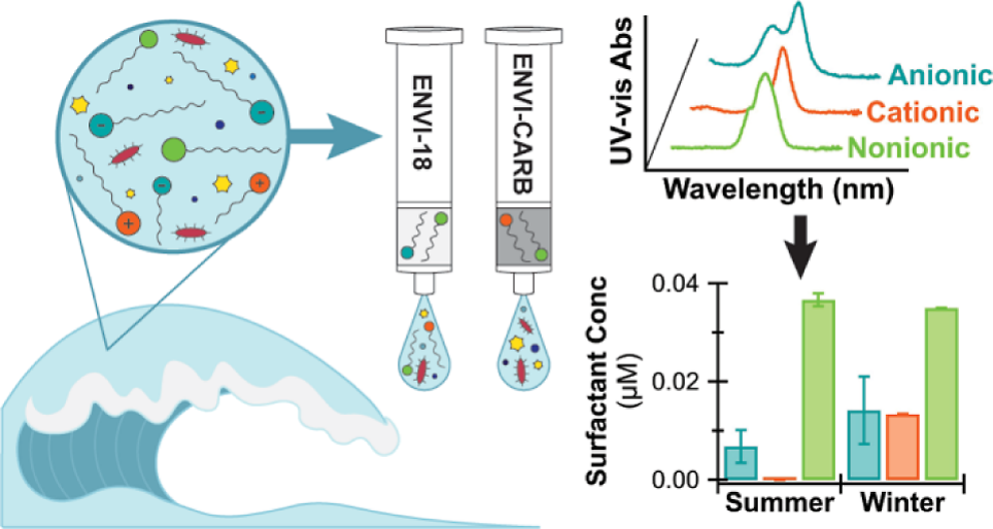

Surfactants are amphiphilic molecules that adsorb to interfaces and affect the interfacial tension. Surfactants in seawater can impact gas-exchange, surface properties, and the composition and fate of sea spray aerosol. The accurate quantification of surfactants and their classes is crucial to constraining the effect of surfactants in seawater and their role in air–sea exchanges. Here, we evaluate and optimize a solid phase extraction (SPE) method paired with colorimetry and UV–vis spectroscopy to quantify the concentrations of anionic, cationic, and nonionic surfactants in seawater. We compare tandem SPE with two-step SPE and different elution volumes and evaluate the impact of different interferents. Improved extraction efficiencies were obtained with an 8 mL acetonitrile elution and with separate ENVI-18 and ENVI-Carb extractions, instead of tandem. With complex surfactant mixtures, the presence of anionic surfactants interfered with the quantification of cationic surfactants and caused underestimations of up to 83%. Using a two-step extraction and analyzing each seawater SPE extract separately during colorimetric quantification help avoid the effects of interferents and ensure more representative quantification of surfactants. With this method, average seawater surfactant concentrations ranged from 0.04 to 0.06 μM. At the highest concentrations, the class composition comprised 23% anionic, 21% cationic, and 56% nonionic surfactants.

## Abstract 2

A targeted two-step solid phase extraction method for surfactants was tested in conjunction with colorimetric UV–vis spectroscopy quantification to better represent and quantify the composition of anionic, cationic, and nonionic surfactants in seawater.

## 1 Introduction

Surfactants are a class of molecules that contribute to the organic composition of seawater and, due to their amphiphilic nature, adsorb to interfaces and concentrate at the ocean surface.^1−3^ This presence of both a hydrophilic head and a hydrophobic tail allows surfactants to play a role in a variety of important environmental processes, including gas exchange^3,4^ and the production of sea spray aerosol (SSA) at the ocean–air interface.^5^ As waves entrain air beneath the ocean surface, surfactants are scavenged by bubbles and brought to the surface of the ocean.^5,6^ Surfactants can accumulate in the sea surface microlayer (SML), which is operationally defined as the top 1–1000 μm of the ocean surface.^5,6^ This organic enrichment at the ocean–air interface dictates the composition and enrichment of surfactants in the emitted SSA.^7−9^ The composition and concentration of surfactants strongly influence the atmospheric fate of SSA due to surfactant adsorption to particle interfaces.^5,10−12^ Surfactants can lower the surface tension of SSA and/or affect their hygroscopicity, thus modulating the ability of SSA to act as cloud condensation nuclei.^10,12−14^ The influence of surfactants on SSA hygroscopicity has been shown to vary as a function of the ionic class.^10,12^ Constraining the chemical composition of marine-derived surfactants is crucial to understanding their effects on air–sea fluxes, influencing SSA chemistry, hygroscopic growth, and cloud activation potential, which altogether determines the contribution of SSA to earth’s radiative balance.

The surfactant composition of SSA is related to the complex composition of the SML and underlying seawater.^15−17^ It is therefore important to understand the types and concentrations of surfactants in seawater. Marine-derived surfactants are often a result of microbial activity and encompass a diverse variety of properties, structures, and molecular weights.^18^ Various species and strains of marine bacteria produce surfactant molecules such as phospholipids, glycolipids, and lipopeptides.^18^ The surfactants’ complexity makes them difficult to study, but previous work has utilized mass spectrometry, tensiometry, and colorimetry to characterize surfactants in seawater and SSA based on their molecular structure, surface activity, and ionic headgroup.^8,19−24^

Solid phase extractions (SPEs) in which specific molecules are selectively extracted by a sorbent material are useful for extracting and concentrating organic molecules and surfactants from complex matrices for further analysis. Colorimetry, in which specific dyes are bound to target surfactant molecules, and quantification with UV–vis spectroscopy have previously been used to selectively determine the concentrations of anionic, cationic, and nonionic surfactants from seawater.^8,25−27^ Surfactants in SPE extracts were complexed with dyes specific to each surfactant class, and the complexes were used to quantify surfactant concentrations with UV–vis spectroscopy.^28^ From a single-step SPE procedure, surfactant concentrations between <0.03 and 0.52 μM were measured in seawater extracts, with the surfactant composition mostly comprising anionic surfactants.^8^ A two-step SPE technique was recently developed to separate surfactants based on their ionic class for mass spectral characterization and formula identification.^19,20^ The surfactant extraction efficiency, and specifically the extraction efficiency of cationic surfactants, was increased by processing seawater through two reversed-phase SPE cartridges.^20^ While the two-step extraction improved the efficiency for mass spectral characterization, which can characterize the broad structural composition of the extracted organic matter, this method needs to be optimized for colorimetry and UV–vis spectroscopy to quantify surfactant concentrations.

This study evaluates the use of the two-step surfactant-targeted SPE with the colorimetric quantification of extracted surfactants for a more comprehensive analysis of anionic, cationic, and nonionic surfactant concentrations in seawater. Both standard solutions and collected seawater were analyzed to understand factors, such as sample matrix interferences and SPE elution types, that influence surfactant extraction efficiency and quantification and to highlight important considerations when these methods are used for seawater. In optimizing the extraction of surfactants from sample matrices, a more accurate representation of the seawater surfactant composition can be achieved to inform the role and influence of surfactants in seawater.

## 2 Methods

In this study, we used solutions of standard surfactants, mock seawater, and collected seawater to test the efficiency of different parameters of surfactant quantification using SPE, colorimetry, and UV–vis spectroscopy. The details of the tests are outlined in the following sections and include quantification of the interference of different surfactant classes, optimization of SPE elution volumes, and comparison of tandem and two-step extractions. While previous studies have measured surfactant concentrations in seawater, the effect of other surfactant classes, as well as other method parameters, on the surfactant quantification has not been studied. The basic method first isolates organic molecules. Then, the organic molecules that complex with the dyes are measured and considered to be surfactants based on the properties necessary to complex to a given dye.

### 2.1 Standard Solutions

Reagent-grade chemicals and ultrapure water (18.2 MΩ·cm) were used to make all calibration and test solutions. All blank solutions are ultrapure water. Exact quantities and concentrations are described later. Purities and vendors are listed in Text S1. These include sodium chloride (NaCl) and glucose to model a simple seawater composition. Surfactants include anionic surfactants sodium dodecyl sulfate (SDS) and dioctyl sodium sulfosuccinate (AOT), cationic surfactants cetyltrimethylammonium salt (CTAC) and benzethonium chloride (Hyamine), and nonionic surfactants polyethoxylate lauryl ether (Brij) and polyoxyethylene dodecyl ether (Genapol). The chosen standard surfactants encompass the range of ionic classes and molecular sizes previously reported for marine surfactants.^8,20,29,30^

### 2.2 SPE and Extraction Efficiency

The surfactant-targeted SPE methods used here are based on those in Burdette and Frossard^20^ utilizing a two-step SPE with ENVI-18 and ENVI-Carb (Text S2). For every SPE, each cartridge was conditioned with 6 mL of acetonitrile (ACN), followed by 12 mL of ultrapure water. Sample volumes extracted were either ∼100 or ∼200 mL. After SPE, each cartridge was fully dried under low vacuum (∼5 psi for ∼1 min), and the retained sample organic fraction was eluted with ACN or acetone. More details on the extraction methods are in Text S2, and specifications for each experiment are in Table S1 and Sections 2.4 and 2.5. Extraction efficiencies were calculated by dividing the measured surfactant concentration by the actual surfactant concentration and multiplying by 100%.

### 2.3 Colorimetric Quantification Method Validation and Matrix Effects

The first test includes a method validation of the colorimetric method and surfactant quantification by UV–vis spectroscopy. Quantification of anionic, cationic, and nonionic surfactants was performed using colorimetry and UV–vis spectroscopy, following previous methods.^28^ Specific details are listed in Text S3. Concentrations of surfactants were chosen to encompass the range previously measured^8^ and to allow for differences in concentrations and extraction efficiencies to be resolved. Two surfactants per surfactant class were tested (Table 1). 25 mL solutions of each surfactant were made at concentrations of 5 μM for the anionic (SDS and AOT) and cationic (CTAC and Hyamine) surfactants and 10 μM for the nonionic surfactants (Brij and Genapol). Then, aliquots of each standard, 1.1 mL for anionic or cationic surfactants and 1.5 mL for nonionic surfactants, were used in the colorimetric method (Text S3). Only dyes specific to the target surfactant class were used. The measured concentration for each standard solution was divided by the actual concentration to determine the percent recovery.

**Table 1:** Average Percent Recoveries for Solutions of Individual Surfactants for Each Class

anionic	percent recovery (%)	cationic	percent recovery (%)	nonionic	percent recovery (%)
SDS	120.3 ± 4.8	CTAC	110.1 ± 15.4	Brij	86.4 ± 1.9
AOT	104.5 ± 2.4	Hyamine	140.7 ± 3.2	Genapol	20.0 ± 1.4^a^

a One of the three replicates were below the limit of detection; two values reported here.

The second test investigates the influence of other classes of surfactants present in the sample matrix on the quantification of an individual surfactant class. 25 mL solutions of individual, target surfactants (SDS, CTAC, or Brij) were made at concentrations of 1 or 5 μM. In each solution, an interferent surfactant (SDS, CTAC, or Brij) was added at high concentration (5 μM, 5× the 1 μM target surfactant concentration), equal concentration (5 μM, equal the 5 μM target surfactant concentration), or low concentration (1 μM, 0.2× the 5 μM target surfactant concentration). Each solution was shaken vigorously for 5 min to ensure complete mixing. Then, aliquots of each, 1.1 mL for anionic or cationic surfactant quantification, and 1.5 mL for nonionic surfactant quantification, were used directly in the colorimetric method (Text S3). Percent differences were calculated using the measured concentration and actual concentration of the target surfactant in the solution with the interfering surfactant class.

### 2.4 SPE Parameters Optimized and Tested with Surfactant Quantification

Several SPE method factors were investigated and compared to assess their effects on measured surfactant concentrations and extraction efficiencies, including sample matrix effects, elution steps, and individual compared to tandem extractions. Individual extractions involved only a single cartridge for a given extraction, while tandem extractions involved sample flow through two stacked cartridges. Additional details on the extraction methods are in Text S2, and specifications for each experiment are in Table S1. Unless otherwise noted, the parameters varied and tested here, such as SPE sorbent material and elution volumes, are based on parameters used in previous methods. The goal in this study is to optimize this extraction method for surfactants in seawater.

The first test focuses on the effect of the SPE elution volume on the surfactant extraction efficiency for each of the two SPE cartridges. A 1 L test solution of 35 g L^–1^ NaCl and a mixture of SDS, CTAC, and Brij, with target hydrated extract concentrations of 1.8, 1.8, and 7 μM, respectively, was used to simulate a seawater matrix. 100 mL aliquots of this solution were filtered (0.45 μm), similarly to seawater samples.^19^ The solutions were then extracted with one cartridge to individually test the surfactant extraction efficiencies. SPE elution volumes of 6, 8, or 12 mL ACN were tested in duplicate extractions of the same parent solution. The ACN extracts were dried, rehydrated in ultrapure water to ∼4 mL, and analyzed for all three surfactant classes with colorimetry and UV–vis spectroscopy (Text S3).

The next test investigates the influence of using the two SPE cartridges in tandem and of the sample matrix on the surfactant extraction efficiency. Surfactant-only solutions were made with a single surfactant standard, and mock seawater solutions were made with 35 g L^–1^ NaCl, 0.01 M glucose, and a single surfactant standard. Each solution had a target hydrated extract concentration of 5 μM for anionic and cationic surfactants and 10 μM for nonionic surfactants. 100 mL was passed through the two cartridges in tandem (ENVI-Carb and then ENVI-18). The cartridges were paired so that the sample not retained in the ENVI-Carb cartridge directly flowed into the ENVI-18 cartridge. After the full 100 mL was processed, the two cartridges were dried individually and eluted with 8 mL of ACN into separate vials. Then, the extracts were dried with pure nitrogen, rehydrated to ∼4 mL with ultrapure water, and analyzed with colorimetry and UV–vis spectroscopy (Text S3).

### 2.5 SPE Methods Compared for Collected Seawater Samples

The final SPE tests used seawater to compare the use of ENVI-18 and ENVI-Carb cartridges in tandem extractions and in separate two-step extractions. Details on seawater collection are in Text S4. These two collected water types are described throughout as North Atlantic (NA) seawater and Delaware Bay (DB) water.

200 mL NA seawater samples were extracted with tandem or two-step SPE, then dried, and rehydrated. The tandem extraction followed the same procedure used on test solutions (Section 2.4), with 200 mL of seawater through both cartridges and an 8 mL ACN elution. For the two-step method, 100 mL went through each cartridge separately, and a 4 mL ACN elution was used. The extracts were then dried with pure nitrogen and rehydrated with ∼4 mL of ultrapure water. The anionic, cationic, and nonionic surfactant concentrations of each of the two extracts, ENVI-18 and ENVI-Carb, for each sample were measured with colorimetry and UV–vis spectroscopy (Text S3).

The same parent solution of DB water was used in several tandem extractions to test the effect of different SPE method modifications on the measured surfactant concentrations, including SPE elution volume, sample filtration, and separate compared to combined ENVI-18 and ENVI-Carb extracts for colorimetric analysis. For all of these tests, the extraction was in the tandem configuration of ENVI-Carb then ENVI-18, following the procedure used in the tandem extractions of standard solutions (Section 2.4 and Text S2).

The elution volume tests involved tandem extractions of 100 mL of DB water with a 4 or 8 mL ACN elution. Each ENVI-18 and ENVI-Carb extract was dried, rehydrated to ∼4 mL in ultrapure water, and analyzed for anionic, cationic, and nonionic surfactants with colorimetry and UV–vis spectroscopy (Text S3).

The sample filtration test involved tandem extractions of 100 mL samples of filtered (0.45 μm) and unfiltered DB water. Each of the two SPE cartridges for each sample was eluted with a series elution of 4 mL of ACN, then 2 mL of ACN/acetone (1:1 v/v), and then 2 mL of acetone. The paired ENVI-18 and ENVI-Carb extract of each sample was then combined into a single vial, dried, and rehydrated to ∼4 mL for anionic, cationic, and nonionic surfactant analysis with colorimetry and UV–vis spectroscopy (Text S3).

The final test evaluated the surfactant quantification of either separated or combined ENVI-18 and ENVI-Carb extracts obtained from tandem extractions of filtered 100 mL aliquots of DB water. Each SPE cartridge was eluted with a series elution, 4 mL of ACN, then 2 mL of ACN/acetone (1:1 v/v), and then 2 mL of acetone. The ENVI-18 and ENVI-Carb extracts were kept separate for three samples and combined into a single vial for two samples. Both the separate and combined extracts were then dried and rehydrated to ∼4 mL for anionic, cationic, and nonionic surfactant analysis with colorimetry and UV–vis spectroscopy (Text S3).

## 3 Results and Discussion

### 3.1 Direct Colorimetric Analysis of Standard Solutions

#### 3.1.1 Colorimetric Method Validation

To test the colorimetric methods and calibration curves used for determining concentrations of unknowns, we measured single surfactant standard solutions. The reported average percent recoveries and standard deviations are the result of at least two to three replicate colorimetric analyses of the same standard solution (Table 1). The percent recoveries are mostly within expected ranges (Table 1 and Figure S4), close to 100% recovery for anionic and cationic surfactants and within the 95% prediction interval of the calibration curves for all surfactant classes (Figures S1–S3). See Text S5 for further discussion of the colorimetric method validation.

#### 3.1.2 Interference from Other Surfactant Classes

The composition of surfactants in seawater from previous studies indicates the presence of multiple classes of surfactants, primarily anionic and nonionic.^8^ While studies utilizing this method have investigated the effect of ionic species on surfactant quantification,^21^ the effect of the presence of other surfactant classes has not been well constrained. Here, the concentration of each class of surfactant was measured individually in solutions with the target surfactant class and the addition of a single interferent surfactant at low, equal, or high concentrations (Section 2.3 and Table S1).

**Figure 1:**
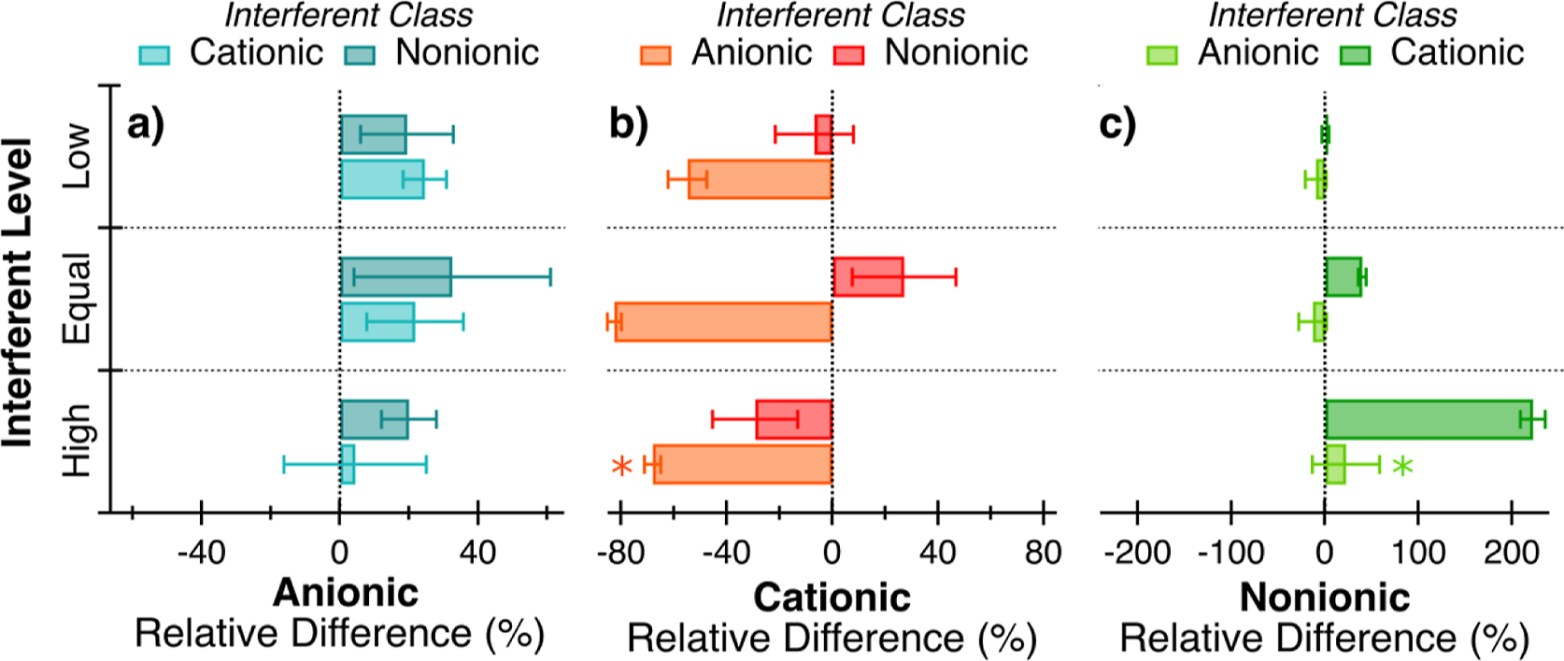
Relative differences in anionic (a), cationic (b), and nonionic (c) surfactants concentrations in solutions with the presence of other classes of surfactant interferents at low (0.2×), equal, or high (5×) concentrations relative to the target surfactant class. The error bars represent the standard deviation of three replicates. Asterisks denote samples with all measured concentrations below method detection limits.

The percent differences of the measured target class concentration to the actual concentration when the target surfactants are in the presence of potential interferent surfactants at different concentrations are shown in Figure 1 and Table S2. The measured concentrations of anionic surfactants were the least affected by the presence of other surfactant classes in solution (Figure 1). All average relative differences were within 33% and there was no considerable difference or trend observed with the interferent class or with increasing interferent concentration (Figure 1a). These measured values align closely with the 21% average percent recovery observed in the quantification of anionic surfactants without sample interferents (Table 1). This shows that even when present in up to five times the anionic surfactant concentration, cationic and nonionic surfactants do not have a major effect on anionic quantification. This may be due to the cationic and nonionic surfactants dampening the electrostatic repulsions of the anionic surfactant headgroups which could facilitate better surfactant-dye complexation.

Conversely, cationic surfactant quantification is negatively impacted by the presence of other classes of surfactants (Figure 1b). For all concentration levels measured, anionic surfactants caused underestimations of up to 83% for cationic surfactants. This was exacerbated by increasing the anionic surfactant concentration. A 5-fold increase of the SDS concentration from low (0.2×) to equal concentrations relative to CTAC caused CTAC to be further underestimated by 15%. At the highest concentration level of added SDS, the measured cationic concentration fell below the detection limit, preventing accurate cationic quantification. If anionic surfactants are present in concentrations even 0.2× that of cationic surfactants in seawater, then cationic surfactants may be significantly undermeasured. Due to the opposing ionic charges, anionic surfactants can interact and bind with cationic surfactants^31−33^ and prevent cationic surfactants from binding with disulfine blue dye.^34^ Interactions between anionic and cationic surfactants would also then be expected to affect anionic quantification; however, this was not observed (Figure 1a). Sodium sulfate used to accelerate phase separation in the anionic quantification method is absent in the cationic colorimetry method.^28,35^ Its addition may reduce electrostatic interactions between anionic and cationic surfactants present in solution and prevent any potential inhibition of anionic–ethyl violet dye complexation and anionic quantification caused by cationic surfactants.

Nonionic surfactants had little to no influence on cationic quantification when present in low and equal levels of Brij relative to CTAC (Figure 1b). For CTAC measured with no interferents (Table 1), percent recovery spanned a range from −5% to 26%. This range includes the percent relative differences seen for CTAC in the presence of the nonionic surfactant Brij (Figure 1b). When present in abundance at five times the cationic concentration, however, nonionic surfactants interfered with cationic quantification and caused an underestimation of about 30% (Figure 1b). This demonstrates that the presence of nonionic surfactants does not interfere with cationic surfactant quantification until the nonionic surfactant concentration is equal to or greater than that of the cationic surfactants. CTAC has previously been shown to form aggregates with nonionic surfactant-like polymers^36,37^ which could be amplified with increasing nonionic surfactant concentrations and interfere with CTAC–disulfine blue dye complexation, liquid extraction, and subsequent quantification.

Anionic surfactant SDS at each of the three concentration levels had little to no effect on the measured nonionic surfactant Brij concentrations (Figure 1c). The standard deviation of the measured nonionic concentration encompasses the true Brij concentration when SDS was present at low, equal, or high concentrations relative to Brij (Figure 1c). This demonstrates that anionic surfactant concentrations up to five times that of nonionic surfactants do not interfere directly with nonionic surfactant quantification. This is likely due to the difference in the way the nonionic dye, cobalt thiocyanate, binds with the polyoxyethylene chain of Brij, which is not present in the anionic surfactant SDS.

The presence of cationic surfactant CTAC caused higher than actual nonionic concentrations to be measured when present in equal or higher concentrations (Figure 1c). Cationic surfactants have been previously reported to cause overestimations in the colorimetric determination of nonionic surfactants.^38−40^ This was confirmed herein, as CTAC in solution may have facilitated complexation of Brij with the cobalt thiocyanate dye by increasing the stabilization of the dye-complex. This nonionic surfactant concentration overestimation increased as the CTAC concentration increased (Figure 1c). At equal concentrations, CTAC caused a 40% overestimation of Brij that then further increased to a 220% overestimation when CTAC was in abundance at five times the concentration of Brij (Figure 1c). This demonstrates that the presence of cationic surfactants may cause overestimations in measured concentrations of nonionic surfactants.

The effects of surfactant interferents on the quantification of the target surfactant class of interest show the potential for overestimations or underestimations depending on the surfactant class composition (Figure 1). The largest deviations were observed for anionic surfactants influencing cationic quantification and cationic surfactants influencing nonionic quantification (Figure 1). Since anionic surfactants can cause such underestimation of cationic surfactants, the separation of these surfactant classes is important for accurate quantification.

Previous work demonstrated that SPE with ENVI-18 isolates mainly anionic and nonionic surfactants, while those with ENVI-Carb isolate mainly nonionic and cationic surfactants.^20^ While both cartridge sorbent materials retain nonionic surfactants, an initial extraction with ENVI-18 may allow for nonionic surfactants to be extracted prior to the extraction with ENVI-Carb. This may minimize nonionic surfactant overestimation caused by the presence of cationic surfactants in the same sample extract. The initial ENVI-18 extraction will also separate anionic surfactants so that they will not interfere with the quantification of cationic surfactants, targeted in the subsequent ENVI-Carb extraction. To mitigate interference from different surfactant classes on the colorimetric analyses of a given surfactant class, each cartridge extract should be analyzed separately.

These results are consistent with previous work quantifying surfactants in seawater and aerosol that exhibited higher fractions of anionic surfactants compared to cationic and nonionic surfactants.^8,21^ This may have been due to the large fraction of anionic surfactants in these samples or underestimation of measured concentrations of cationic surfactants. Cationic surfactants were not measured in seawater samples previously analyzed with this method,^8^ potentially due to the interference of anionic surfactants present.

### 3.2 SPE of Standard Surfactant Solutions

#### 3.2.1 Optimizing SPE Elution for Improved Surfactant Extraction

A key component to improving the SPE extraction efficiency is optimizing the elution of the retained target compounds. This includes the choice of elution volume and solvent.^41^ Details of this experiment are in Section 2.4 and Table S1. The original SPE method produced high extraction efficiencies of surfactants from each surfactant class based on standard surfactant concentrations measured with mass spectrometry.^20^ That method included a two-step SPE with ENVI-18 and ENVI-Carb cartridges and 4 mL ACN for each elution.^20^

**Figure 2:**
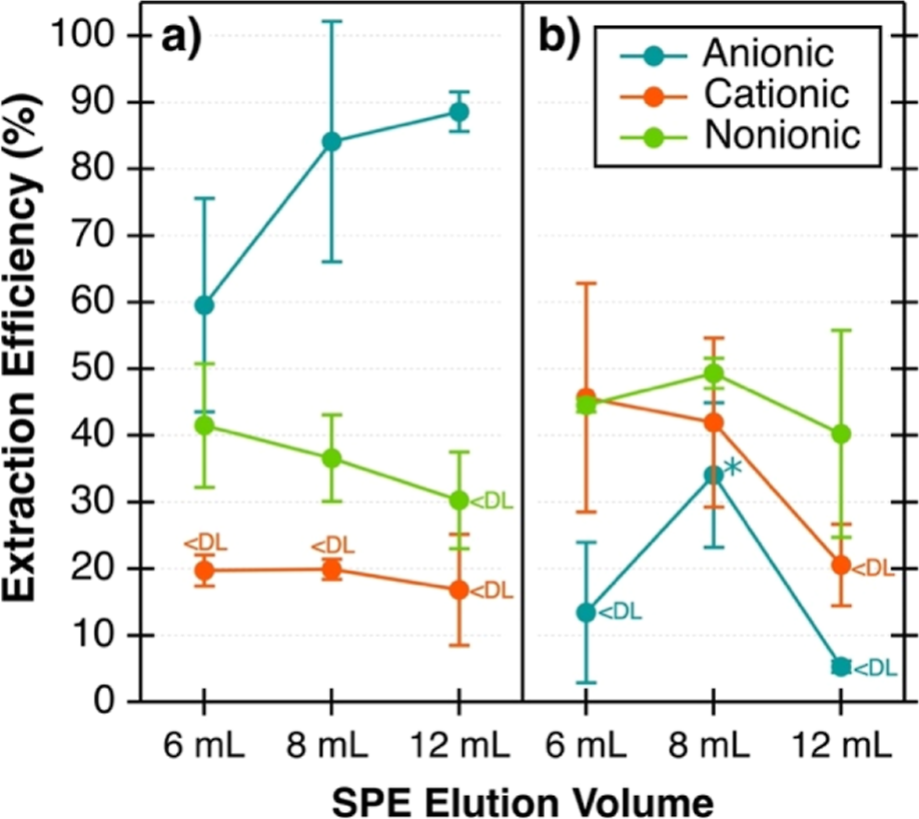
Extraction efficiencies of anionic, cationic, and nonionic surfactants measured from (a) ENVI-18 and (b) ENVI-Carb SPE extracts of the 35 g L^–1^ NaCl solution containing SDS, CTAC, and Brij. The error bars represent the standard deviation of at least two extraction replicates. Measured surfactant concentrations below detection limit are designated by <DL. The asterisk denotes a single extraction (and single colorimetry sample) with error bars instead representing the 95% confidence interval.

Figure 2 shows the extraction efficiencies of anionic, cationic, and nonionic surfactants for each SPE cartridge as a function of different elution volumes. The overall trends in surfactant extraction efficiencies matched those from the original method, with anionic surfactants extracted more efficiently with ENVI-18 cartridges and cationic surfactants extracted more efficiently with ENVI-Carb cartridges.^20^ Similarly, nonionic surfactants were extracted and measured from both ENVI-18 and ENVI-Carb extractions.

When comparing the extraction efficiencies measured with colorimetry to those obtained previously with mass spectrometry,^20^ however, the extraction efficiencies are notably lower for nonionic and cationic surfactants (Table S3). Previously reported ENVI-Carb extraction efficiencies were 75–77% for cationic surfactants, whereas those measured here were only 21–46% (Figure 2). For nonionic surfactants extracted with ENVI-18, the previously reported extraction efficiencies were between 63 and 70%, whereas those measured here were only 30 to 42% (Figure 2). Anionic surfactants were the only class with comparable extraction efficiencies. The anionic extraction efficiencies reported previously were 83 to 99% which overlap with the 60 to 89% extraction efficiencies determined by colorimetry shown here (Figure 2). The previously reported extraction efficiencies were calculated with mass spectrometry,^20^ which identifies the specific standard surfactant *m*/*z* but is unable to quantify concentrations of unknown surfactants. Meanwhile, the colorimetry method is a bulk analysis that can be influenced by the presence of other species (Section 3.1.2). Since the test solution used here included a mixture of three classes of surfactants, the measured concentrations and thus calculated extraction efficiencies of a given surfactant class may be impacted by the presence of other surfactant classes. These factors explain why the extraction efficiencies of certain surfactant classes are lower than those obtained previously with mass spectrometry.

The optimal elution volume for both ENVI-18 and ENVI-Carb is 8 mL of ACN based on this volume yielding higher extraction efficiencies for the three surfactant classes (Figure 2). With 8 mL elution volumes, the average extraction efficiencies are 84% and 37% for anionic and nonionic surfactants, respectively, with the ENVI-18 cartridge (Figure 2a) and 49% and 42% for nonionic and cationic surfactants, respectively, with the ENVI-Carb cartridge (Figure 2b). With an increase in the ACN elution volume from 6 to 8 mL, average anionic surfactant extraction efficiencies were increased by 24% in the ENVI-18 extraction and average nonionic surfactant extraction efficiencies were increased by 5% in the ENVI-Carb extraction. Cationic surfactants were not impacted. This was shown by similar cationic surfactant extraction efficiencies of 46 ± 17% for 6 mL of ACN and 42 ± 13% for 8 mL of ACN for the ENVI-Carb extractions. No further improvement was observed for any surfactant class by increasing the elution volume from 8 to 12 mL (Figure 2). In some cases, it caused a decrease in the average surfactant extraction efficiency, as shown for nonionic surfactants in ENVI-18 extractions and both nonionic and cationic surfactants in ENVI-Carb extractions (Figure 2). This was observed for cationic surfactants from the ENVI-Carb extraction that fell below the detection limit with a 12 mL ACN elution. This shows that using higher elution volumes (12 mL) could cause surfactant loss. This is unexpected but may occur with increased adsorption of surfactants to the vial walls during the solvent evaporation step, where the larger volume means more contact with the surface of the vial and a longer time for evaporation.^42^

#### 3.2.2 Tandem SPE of Surfactants and Sample Matrix Effects

The original surfactant SPE method involves two separate extractions of a single sample, requires significant time, and may not be efficient for large sample sets. Tandem SPE can be more efficient by combining two extractions with two cartridges connected in tandem through which the sample is extracted at the same time. The extraction efficiency of individual surfactants extracted through the tandem extraction of ENVI-Carb into ENVI-18 was compared across 100 mL single surfactant standard solutions and 100 mL mock seawater solutions. The order of the cartridges was chosen so that ENVI-Carb, which has a larger pore size and thus faster sample elution rate, would be first and feed into ENVI-18 to prevent the ENVI-18 sorbent from drying out. Details of this experiment are in Section 2.4 and Table S1.

**Figure 3:**
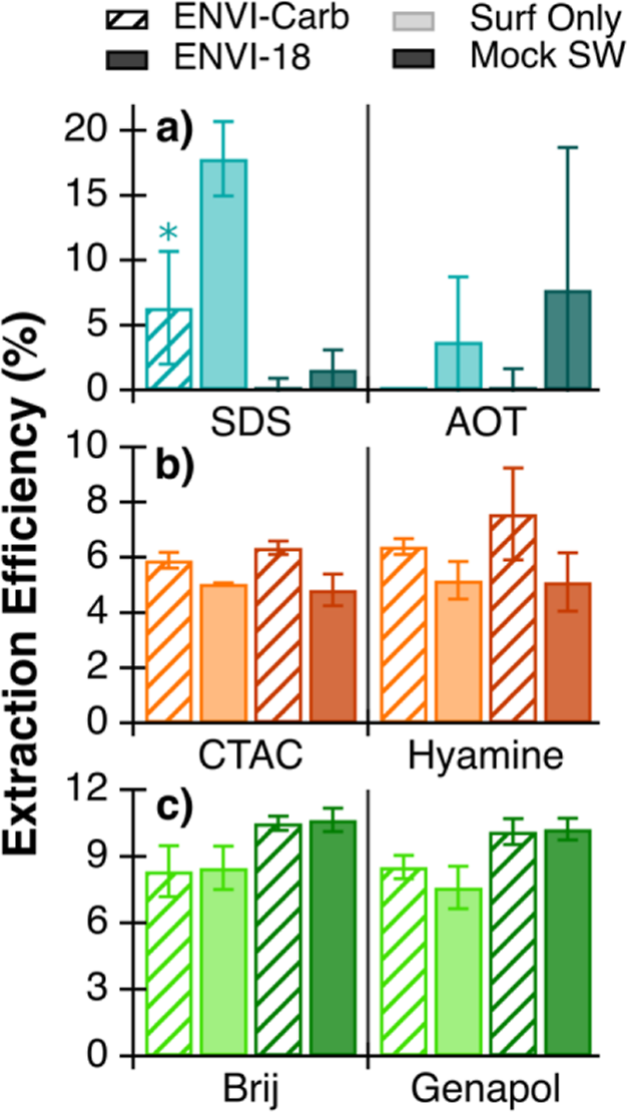
Extraction efficiencies of anionic (a), cationic (b), and nonionic (c) surfactants from tandem SPE (ENVI-Carb into ENVI-18) of surfactant-only solutions (Surf Only, left 2 bars) and surfactants in mock seawater solutions (Mock SW, right 2 bars). The error bars represent the standard deviation of at least two replicate extractions. The asterisk denotes a single extraction with error bars instead representing the 95% confidence interval.

The tandem extractions show very low extraction efficiencies (averages of <20%) across all surfactant classes (Figure 3), which was unexpected in surfactant-only solutions, considering the absence of any other inorganic or organic interferents in solution, and when compared to surfactant extraction efficiencies in the previous tests (Figure 2). For example, with individual extractions, the anionic surfactants had an average extraction efficiency of ∼85% (Figure 2). Using the tandem extraction, this efficiency was then greatly reduced to <20% (Figure 3). For the ENVI-Carb extracts, average surfactant extraction efficiencies decreased by 30% for anionic, 36% for cationic, and 41% for nonionic surfactants when compared to the 8 mL elution extraction efficiencies. Also, the average surfactant extraction efficiency decreased by 60% for anionic and 28% for nonionic surfactants for ENVI-18 extracts.

This low extraction efficiency is unexpected but may be due to the order of cartridges with ENVI-Carb prior to ENVI-18. During the tandem SPE, the flow through the cartridges was very slow, and the rate of extraction varied sample-to-sample, suggesting that some samples may have had better exposure of the target surfactants to the sorbent material. The tandem SPE method is also more complicated than single extractions with separate cartridge conditioning, tandem sample loading, and separate elutions. This complexity and impact on sample flow rate introduces the possibility of more variation and error which can affect SPE efficiency and reproducibility.^43^

Because ENVI-Carb has low extraction efficiencies for anionic surfactants, it was assumed that most anionic surfactants would pass through the ENVI-Carb cartridge without being retained, which was not observed here. This implies that anionic surfactants may be retained in the ENVI-Carb cartridges and that the elution (8 mL of ACN) is not sufficient to break anionic surfactant interactions with the ENVI-Carb sorbent. Previous methods carried out the extractions in reverse with the ENVI-18 extraction first, where the sample that passed through the ENVI-18 cartridge was collected and then later extracted with the ENVI-Carb cartridge.^19,20^ The extraction order here may alter the retention of anionic and nonionic surfactants, causing decreases in extraction efficiencies.

Comparing the surfactant-only solutions (containing single surfactants) to the mock seawater solutions (containing single surfactants, glucose, and NaCl) shows the impact of the sample matrix on the extraction and colorimetric method quantification (Figure 3). These solutions have target extract concentrations of 5 μM each for anionic and cationic surfactants and 10 μM for nonionic surfactants. For nonionic surfactants, the mock seawater has 2% higher extraction efficiencies than the surfactant-only solutions for both SPE cartridges (Figure 3c). The extraction efficiencies for the cationic surfactants were very similar for both sample types and SPE cartridges (Figure 3b), falling within the standard deviation. The effect of the sample matrix was not easily observed due to the overall low measured cationic concentrations (Figure 3b). Meanwhile, the extraction efficiencies for the anionic surfactants showed no consistent trends (Figure 3a). The ENVI-18 extraction efficiency of SDS decreases 16%, while AOT’s extraction efficiency increases 7% in the presence of salt and glucose (Figure 3a).

Salt in solution and increased organic loading from glucose may affect how surfactants are retained and extracted with the SPE sorbent.^43−46^ However, it is unclear why the presence of salt would increase the extraction efficiency, as shown with a slight increase for the nonionic surfactants (Figure 3c). This may be due to the ionic strength of the solution, which could affect the retention mechanism of nonionic surfactants with the reversed phase sorbents. If the other components of mock seawater affect the ENVI-18 extraction efficiency, this indicates that either glucose is not all retained in the first SPE (ENVI-Carb) and is interfering with the retention or quantification of surfactants from the ENVI-18 extraction, or the salt in the sample matrix is affecting surfactant retention and extraction with ENVI-18. Colorimetric measurements of the glucose-only solution confirmed that glucose is not falsely quantified as a surfactant, and the results matched those of pure water blanks (Table S4).

These tandem SPE experiments show that salt and glucose may influence surfactant extraction and quantification, and more studies are needed. This could bias reported concentrations of surfactants by either underestimating or overestimating depending on the complexity of the sample matrix. This effect could be different depending on the organic loading of the seawater sample and the sorbent material used. Overall, this demonstrates that tandem extractions with ENVI-Carb, followed by ENVI-18 have lower surfactant extraction efficiencies than individual extractions for both surfactant-only solutions and model seawater.

### 3.3 Concentrations and Ionic Composition of Surfactants in Environmental Samples

#### 3.3.1 Comparing Tandem and Separate Two-Step Extractions of Seawater

Tandem extractions with 8 mL of ACN elution were also performed on collected NA seawater samples and compared to samples extracted with the original two-step SPE method using 4 mL of ACN elution. Experimental details are in Section 2.5 and Table S1. Measured concentrations are the sums of the surfactant concentrations from the two cartridges. The limit of detection was lowered for this sample set (Text S3).

**Figure 4:**
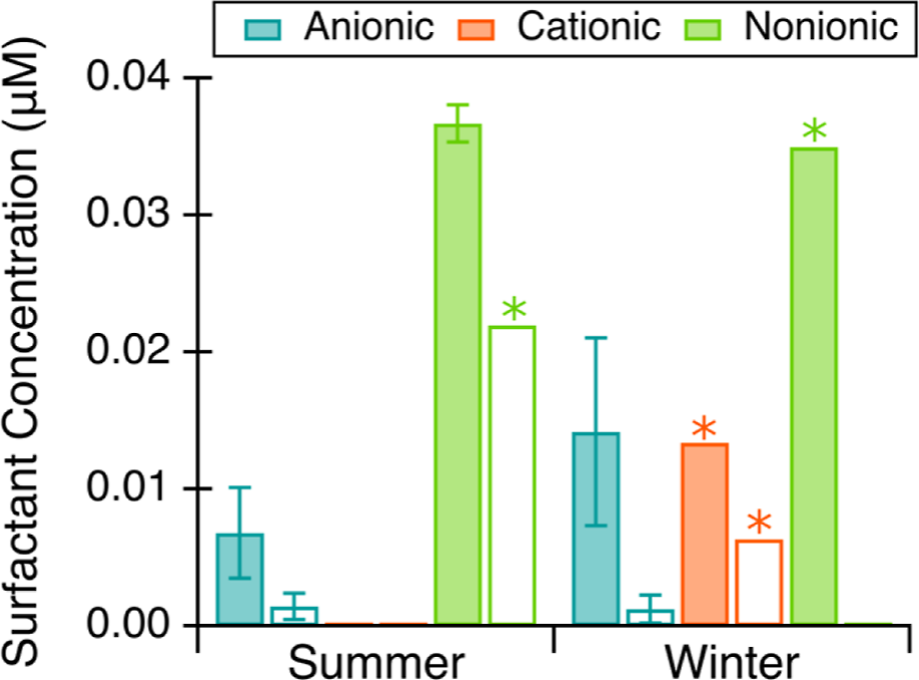
Average seasonal NA seawater surfactant concentrations for extracts from the two-step extraction (solid bars) and tandem ENVI-Carb to ENVI-18 extraction (empty bars). Error bars represent the standard deviation of at least two replicate extractions. Asterisks denote instances where bars represent the only sample with measured concentrations above the detection limit.

The measured concentrations of all surfactants obtained through tandem SPE were lower than those extracted with the original two-step extraction method even though the tandem extraction used a greater ACN elution volume (Figure 4). Surfactant concentrations were lower by up to 0.013, 0.007, and 0.035 μM for anionic, cationic, and nonionic surfactants, respectively, with tandem SPE. Since the NA seawater had a low organic content (Text S4), the majority of surfactant concentrations for the tandem SPE extracts were not above the detection limit (Figure 4). Here, we show that a two-step extraction has higher extracted surfactant concentrations compared to the tandem extraction in NA seawater, implying that the two-step method should be used for seawater samples.

For samples extracted with the two-step extraction, differences in the surfactant composition between NA seawater collected during summer and winter were identified. Anionic surfactants were present in higher concentrations in the winter, but the percent of anionic surfactants out of the total surfactant composition was relatively consistent, increasing from 16 to 23% from summer to winter (Figure 4). Cationic surfactants were undetected in the summer but measured above the detection limit for one winter sample, comprising 21% of the total surfactants (Figure 4). Nonionic surfactant concentrations were comparable between summer and winter, with no obvious seasonal trend. In the winter sample with the measured cationic concentration above the detection limit, nonionic surfactants were 56–84% of the total seawater surfactant composition (Figure 4). The seasonal trends and surfactant composition identified from samples extracted with the two-step extraction were not observed for samples extracted with the tandem SPE method.

The total average surfactant concentration was 0.043 μM for summer seawater and 0.063 μM for winter seawater (Figure 4). These concentrations fall within the lower range of those previously measured for NA seawater utilizing the same colorimetric quantification methods (<0.03 to 0.52 μM).^8^ Anionic surfactants were the dominant class previously, but here, the dominant class is nonionic surfactants (Figure 4). The differences in surfactant composition can point to differences in the extraction methods. Previous studies only used a single-step SPE extraction (with C18 sorbent); however, the two-step SPE extraction allows for the extraction of cationic and nonionic surfactants. This improved extraction is reflected by the higher abundance of nonionic surfactants and the presence of cationic surfactants, which previously went undetected.

#### 3.3.2 Testing SPE Method Variations with DB Water

To test whether improvements in surfactant extraction and quantification with tandem SPE could be made, different method variations were tested using the same parent solution of collected DB water. Higher concentrations of surfactants have been measured in coastal sites due to anthropogenic activity,^47^ and DB water was selected for these studies due to higher expected surfactant concentrations. The effect of elution volume, sample filtration, and colorimetric analysis on separate versus combined ENVI-18 and ENVI-Carb extracts was explored. The limit of detection was lowered for this sample set (Text S3). Further details are given in Section 2.5 and Table S1.

**Figure 5:**
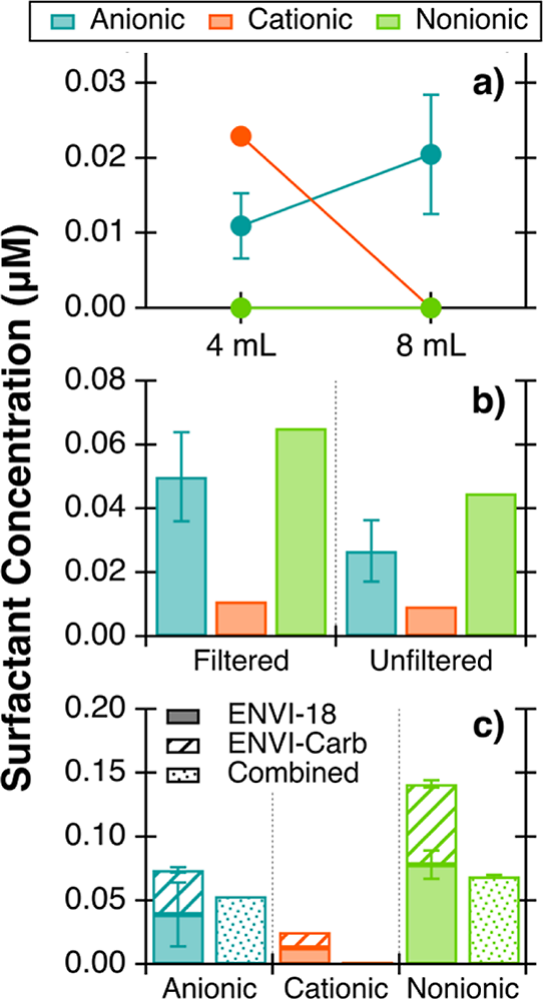
Total surfactant concentrations in DB water from tandem extractions comparing different method conditions including (a) 4 or 8 mL ACN elution, (b) filtered or unfiltered water, and (c) colorimetric analysis on combined or separate ENVI-18 and ENVI-Carb extracts. For (b,c), the elution was a series with 4 mL of ACN, then 2 mL of ACN/acetone (1:1 v/v), and then 2 mL of acetone. Error bars represent the standard deviation of at least two samples. If error bars are not included, that indicates only one measured concentration above the detection limit.

The 4 and 8 mL ACN elution volumes were compared to see if an increase in elution volume improved extraction efficiencies and whether the extracted surfactant class composition was affected (Figure 5a). The increase in average anionic concentration from 0.011 μM with 4 mL of ACN to 0.020 μM with 8 mL of ACN (Figure 5a) is consistent with the improvement in anionic surfactant extraction of standard solutions (Figure 2a). Nonionic surfactants were undetected for both elution volumes, but cationic surfactant concentrations were 0.023 μM from extracts eluted with 4 mL of ACN and undetected with 8 mL of ACN (Figure 5a). Since ENVI-18 and ENVI-Carb extracts were combined for these analyses, the increase in anionic surfactant concentration and absence of measured cationic surfactants for extracts using the increased 8 mL SPE elution volume suggest that the higher quantity of anionic surfactants in the SPE extracts interferes with cationic quantification (Figure 1b).

While anionic concentrations measured here were higher than those measured in NA seawater (Figure 4), the measured surfactant concentrations in DB water were still low compared to previous studies^8^ and lacked nonionic surfactants (Figure 5a). Based on these results and those from testing different elution solvent compositions (Table S5 and Figure S5), the SPE elution step for each cartridge was altered to 4 mL of ACN, 2 mL of ACN/acetone (1:1 v/v), and 2 mL of acetone for the remaining tests (Figure 5b,c) to facilitate more efficient elution of all surfactant classes.

Filtration was also studied to determine if filtering caused an observable loss of surfactants or a selective loss of certain classes. It has been reported that surfactants in environmental samples may be lost during filtration through adsorption onto filter membranes.^48^ The results in Figure 5b suggest that sample filtration prior to SPE actually facilitates better quantification of surfactants. Anionic and nonionic surfactants measured from filtered samples exhibited about 0.02 μM higher concentrations than those from unfiltered samples. Cationic surfactants were, however, not present in sufficient concentrations to identify differences between unfiltered and filtered samples. Filtering removes particulate matter that may interfere with surfactant retention on the SPE sorbent and/or restrict the sample SPE flow rate.^49,50^ Despite the differences in measured surfactant concentrations, the class composition was similar across all three classes for both filtered and unfiltered samples, with 33–39% anionic, 9–12% cationic, and 52–55% nonionic surfactants (Figure 5b).

Since some NA and DB samples exhibited surfactant concentrations below the detection limit, combining both ENVI-18 and ENVI-Carb extracts for colorimetric analysis was tested to increase measured surfactant concentrations. With the two extracts combined into one vial, measured concentrations decreased for all three surfactant classes and cationic surfactants were undetected (Figure 5c). Even the ENVI-18 extract alone had anionic surfactant concentrations within the standard deviation and higher measured cationic and nonionic concentrations compared to those of the combined ENVI-18 and ENVI-Carb extracts (Figure 5c). This confirms the interference of other surfactant classes, as observed with the standard solutions (Figure 1). While separate analyses on two extracts increases sample workload, it is necessary to prevent these interferences which underestimate surfactant concentrations and misrepresent the seawater surfactant composition.

Another key result is the improvement in surfactant extraction obtained when using a different SPE solvent elution composition. Unlike the 8 mL ACN extracts, the combined 4 mL ACN, 2 mL ACN/acetone (1:1 v/v), and 2 mL acetone elution extracts measured concentrations of cationic and nonionic surfactants above the detection limit with tandem SPE. This elution also extracted 60 to 72% higher concentrations of anionic surfactants compared to the 8 mL ACN elution (Figure 5). This shows the potential benefits of using different polarity solvents to elute in a gradient, which could allow for a broader distribution of surfactants to be eluted.

## 4 Conclusions

In utilizing an optimized two-step SPE of seawater, a more comprehensive surfactant composition was measured through colorimetric quantification. Here, we observed nonionic surfactants to be the most abundant class in seawater and comprise up to 55–84% of the total surfactants. Cationic surfactants had concentrations around 0.01 μM in seawater, comprising up to 21% of surfactants. Both of these results are in contrast to previous work that primarily observed anionic surfactants. The second SPE extraction, with ENVI-Carb, allowed for a fraction of surfactants unretained in the ENVI-18 extraction, mainly cationic and nonionic surfactants, to be isolated and quantified, thus demonstrating a major improvement over previous methods.

Improvements to this surfactant quantification method include the use of two SPE cartridges, ENVI-18 and ENVI-Carb, and an increased ACN elution volume (8 mL). Based on standard testing, 8 mL of ACN was the optimal SPE elution volume, improving the extraction efficiencies of anionic and nonionic surfactants by 23% and 5%, respectively, when compared to the 6 mL ACN elution.

It is important, however, to consider underestimations of cationic surfactants (up to 83%) caused by anionic surfactants in the matrix of the sample extract, which had previously not been studied. This, combined with low surfactant concentrations measured from DB water when the two SPE extracts were combined for analysis, suggests that the ENVI-18 and ENVI-Carb extracts should be analyzed separately with colorimetric methods to minimize the impact of interferents. Since the tandem extraction of ENVI-Carb into ENVI-18 can greatly underestimate surfactant concentrations, it is recommended to use the separate two-step extraction to ensure that measured concentrations are representative.

Overall, the recommended surfactant quantification method includes a two-step SPE extraction first through ENVI-18 and then ENVI-Carb, with both cartridges eluted with 8 mL of ACN, and each separate SPE extract analyzed individually with the anionic, cationic, and nonionic colorimetric methods.

Further testing and optimization of the tandem SPE method, including reversing the order of the SPE cartridges and changing the elution solvent compositions, could be performed. Future work could couple supplemental analyses with techniques such as mass spectrometry to investigate how different organic compounds and salinity affect the extraction efficiency of standard surfactants. Continued testing and insights into the extraction of surfactants will improve the isolation and efficient extraction of surfactants of different classes from complex seawater matrices.

## Supporting Information Available

Chemicals, vendors, and purities; specifications of the SPE; individual parameters of the SPE tests; colorimetry and UV–vis spectroscopy method; calibration curve for the anionic surfactants; calibration curve for the cationic surfactants; calibration curve for the nonionic surfactants; seawater and bay water collection; results of the method validation; percent recovery of standard surfactants; percent differences for the surfactant class interference testing; extraction efficiencies of surfactants in salt solutions; measured surfactant concentrations of glucose solution and blank; SPE elution compositions tested; and extraction efficiencies of the anionic, cationic, and nonionic surfactants for different elution compositions with both cartridges (PDF)
