# Supplementary material for: Evaluating the Extraction and Quantification of Marine Surfactants from Seawater through Solid Phase Extraction and Subsequent Colorimetric Analyses
Source: ACS ES T Water. 2024 Oct 25;4(11):4836–46. doi: 10.1021/acsestwater.4c00497 (PMC11555682; doi:10.1021/acsestwater.4c00497)
Supplement: Supplementary file 1 — ew4c00497_si_001.pdf [file ew4c00497_si_001.pdf]

Supporting Information

Evaluating the extraction and quantification of marine surfactants from seawater through solid phase extraction and subsequent colorimetric analyses

Rachel L. Bramblett[1] *and Amanda A. Frossard*[1*]
[1]Department of Chemistry, University of Georgia, Athens, GA, 30606, USA

[*]Corresponding author: Amanda A. Frossard, afrossard@uga.edu

Number of pages: 12

Number of tables: 5

Number of figures: 5

Number of texts: 5

Text S1. List of Chemicals

Chemicals include: sodium chloride (NaCl, ACS grade, Fisher Scientific); glucose (D-(+)-glucose, ≥99.5%, Sigma-Aldrich); various surfactants (Sigma-Aldrich), including anionic surfactants sodium dodecyl sulfate (SDS, ≥99%) and dioctyl sodium sulfosuccinate (AOT, ≥97%), cationic surfactants cetyltrimethylammonium salt (CTAC, 25 wt % in water) and benzethonium chloride (Hyamine 1622, herein called Hyamine), and nonionic surfactants polyethoxylate lauryl ether (Brij 35, herein called Brij) and polyoxyethylene dodecyl ether (Genapol C-100, herein called Genapol); acetonitrile (ACN, ≥99.9% HPLC grade, Fisher Scientific), acetone (≥99.5% ACS grade, Sigma-Aldrich).

Text S2. Solid Phase Extraction Specifications

Each sample was loaded onto the SPE cartridge with pre-cleaned glass syringes and pre-cleaned stainless-steel needles. Only gravity flow (no vacuum) was used to maintain a low sample flow rate and maximize sample interaction with the cartridge sorbent material. After sample throughput was complete, a small volume (2 mL for 100 mL samples and 4 mL for 200 mL samples) of ultrapure water was used to rinse both the sample bottle and syringe. This wash was added to the sample's SPE cartridge and then a final 12 mL rinse of ultrapure water was added directly to each cartridge to fully elute any unretained compounds. The retained compounds were eluted with solvent into pre-cleaned glass vials and then fully dried under nitrogen gas. ENVI-18 (0.5 g bed weight C18 sorbent, Supelco) and ENVI-Carb (0.5 g bed weight graphitized carbon sorbent, Supelco) extracts were always eluted and analyzed separately unless otherwise specified. Both cartridges are reversed-phase SPE based on Van der Waals and dispersion force retention mechanisms. Vacuum was only applied on the extraction manifold when the cartridge needed to be completely dried out after the final wash step and then again after the final solvent elution. Dried extracts were stored at 40°C until colorimetric analysis, at which point they were rehydrated with 3.84 mL of ultrapure water.

For every tandem extraction, the sample was loaded and eluted through ENVI-Carb directly into ENVI-18. This order of cartridges was chosen based on the different flow rates through the different cartridges (ENVI-Carb had a faster flow rate) and the need to prevent cartridges from drying out during the extraction process. In preparation for the tandem extraction, the cartridges were each conditioned separately with 6 mL ACN and 12 mL ultrapure water, and then fixed in tandem with SPE cartridge adapters for sample loading. During the final wash steps, a 2 mL ultrapure water bottle wash and subsequent 6 mL wash of ultrapure water were added directly to the cartridges in tandem. Then the cartridges were separated, and a final 6 mL wash of ultrapure water was added to each cartridge individually to fully elute any unretained compounds. ENVI-18 and ENVI-Carb extracts were always eluted and analyzed separately unless otherwise specified.

Table S1. Experiment specifications for solid phase extraction method testing (SPE tests only)

**Test Description**	**Sample Composition**	**Sample Extract Surfactant Concentrations**	**Sample Filtration**	**Sample Volume (mL)**	**Type of SPE Extraction** Tandem (EC into E18) or Separate (E18 then EC)	**SPE Cartridge Elution**
Colorimetry Method Validation	Single surfactant solutions	5 µM for anionic or cationic*; 10 µM for nonionic*	none	N/A	N/A	N/A
Interference from Other Surfactant Class	SDS + CTAC<br>SDS + Brij 35<br>CTAC + Brij 35	1 µM target, 5 µM interferent*; 5 µM target, 5 µM interferent*; 5 µM target, 1 µM interferent*	none	N/A	N/A	N/A
Increasing ACN Elution Volume	35 g/L NaCl, 1.8 µM SDS, 1.8 µM CTAC, & 7 µM Brij 35	1.8 µM SDS, 1.8 µM CTAC, & 7 µM Brij 35	0.45 µm	100 mL	Separate	6, 8, or 12 mL ACN
Tandem SPE – Surfactant Only vs Mock Seawater	Single surfactant solutions or mock seawater (35 g/L NaCl, 0.1 M glucose, & one surfactant)	5 µM for anionic or cationic; 10 µM for nonionic	0.45 µm	100 mL	Tandem	8 mL ACN
Separate vs Tandem Extraction	North Atlantic subsurface seawater (~5 m)	N/A	0.45 µm	200 mL	Separate & Tandem	4 mL ACN for separate; 8 mL ACN for tandem
Tandem SPE - Different SPE Elution Volumes	Delaware Bay subsurface water (< 1 m)	N/A	0.45 µm	100 mL	Tandem	4 or 8 mL ACN
Sample Filtration	Delaware Bay subsurface water (< 1 m)	N/A	0.45 µm and unfiltered	100 mL	Tandem	4 mL ACN, 2 mL ACN/acetone (1:1 v/v), 2 mL acetone
Separate or Combined SPE Extracts for Colorimetry Analysis	Delaware Bay subsurface water (< 1 m)	N/A	0.45 µm	100 mL	Tandem	4 mL ACN, 2 mL ACN/acetone (1:1 v/v), 2 mL acetone

*These samples are not actual extracts that underwent solid phase extraction but rather test solutions simulated as extracts and treated as such for colorimetric analysis

Text S3. Colorimetric Determination with UV-vis Spectroscopy

Quantification of anionic, cationic, and nonionic surfactants was performed using colorimetry and UV-vis spectroscopy, following previous methods.[1] Samples analyzed with colorimetry were either standard solutions or seawater SPE extracts rehydrated to ~4 mL with ultrapure water. Each sample was split into three aliquots to perform colorimetric analyses for each of the three surfactant classes. Colorimetric reagents added to aqueous samples facilitate surfactant-dye complexation with dyes specific to each surfactant class. Ethyl violet dye complexes with anionic surfactants, disulfine blue complexes with cationic surfactants, and cobalt thiocyanate complexes to the polyoxyethylene oxide chain of nonionic surfactants.[1, 2] To facilitate dye complexation and extraction, acetate buffer, ethylenediaminetetraacetic acid, and sodium sulfate solutions are added to the solutions targeting anionic surfactants, and an acetate buffer is added to the solutions targeting cationic surfactants.[1, 2] The formed surfactant-dye complex is extracted into organic solvent, chloroform for nonionic and cationic surfactant-dye complexes and toluene for anionic surfactant-dye complexes, through a liquid-liquid extraction. The absorbance of the organic layer is then measured with UV-vis spectrophotometry.

Calibration curves, shown in Figure S1-S3, were made from surfactant standards of known concentrations to define the relationship between surfactant concentration and the measured absorbance at a peak wavelength for the surfactant-dye complex. All collected UV-vis spectra were baseline subtracted using the spectra of the pure organic solvent specific to the analysis being performed, toluene for anionic surfactants and chloroform for cationic and nonionic surfactants. The absorbance peaks used for quantification were 612 nm for anionic, 628 nm for cationic, and 621 nm for nonionic surfactants. For each sample, at least three UV-vis spectra were collected and averaged.

The limit of detection for each surfactant class and its corresponding colorimetric method was defined as the mean plus 3.3 times the standard deviation ($\bar{x} + 3.3\sigma$) of the measured absorbance of the colorimetric method blank. Certain sample sets, as noted in the text, with measured concentrations that fell below the defined detection limit utilized a modified detection limit with one standard deviation ($\bar{x} + 1\sigma$) to extrapolate trends and data interpretations.

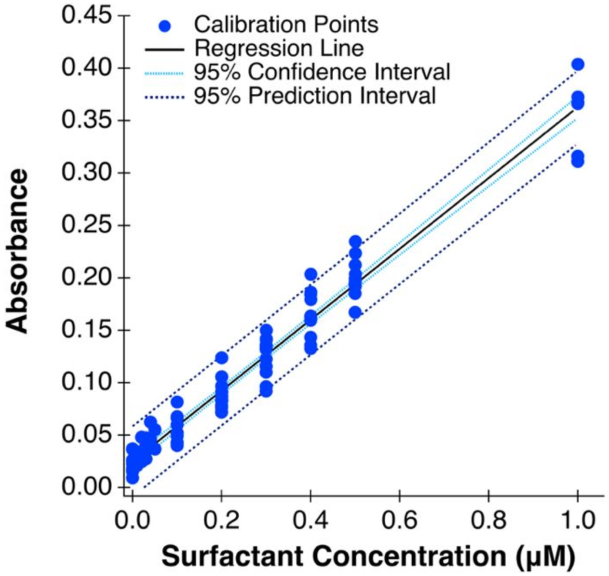

Figure S1. Anionic surfactant calibration curve of AOT and SDS standards with the 95% confidence interval and 95% prediction interval. Absorbances correspond to the measured absorbance at the 612 nm peak for different anionic surfactant concentrations. The equation of the linear regression line is A= 0.338C+0.0249, where A is absorbance and C is surfactant concentration, with $r^2 = 0.965$. The linear regression line includes both AOT and SDS. The limit of detection is 0.117 µM.

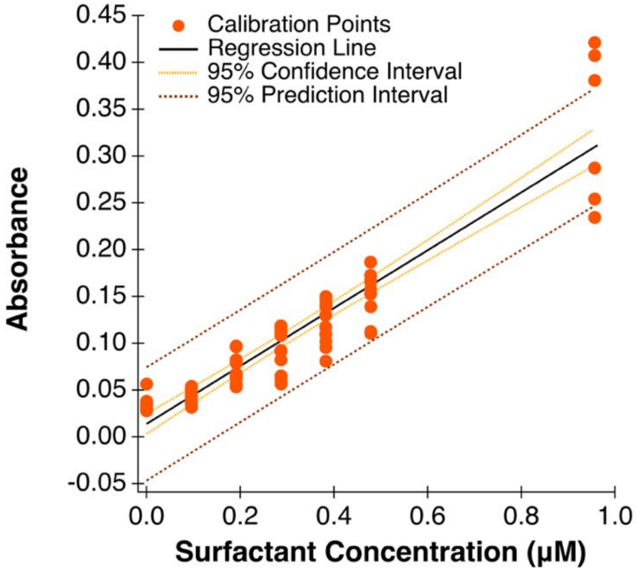

Figure S2. Cationic surfactant calibration curve for CTAC and Hyamine standards with the 95% confidence interval and 95% prediction interval. Absorbances correspond to the measured absorbance at the 628 nm peak for different cationic surfactant concentrations. The equation of the linear regression line is A= 0.309C+0.0139, where A is absorbance and C is surfactant concentration, with $r^2 = 0.872$. The linear regression line includes both CTAC and Hyamine. The limit of detection is 0.094 µM.

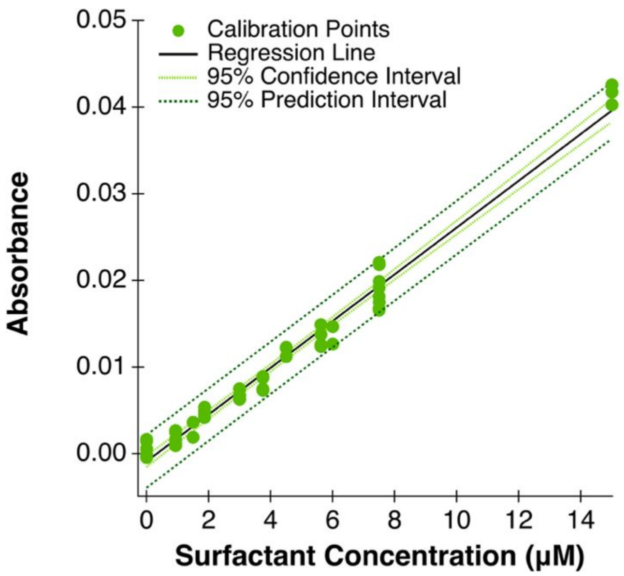

Figure S3. Nonionic surfactant calibration curve for Brij 35 standard with the 95% confidence interval and 95% prediction interval. Absorbances correspond to the measured absorbance at the 621 nm peak for different nonionic surfactant concentrations. The equation of the linear regression line is A= 0.0027C+0.0009, where A is absorbance and C is surfactant concentration, with r^2 = 0.980. The limit of detection is 1.59 μM.

Text S4. Seawater and Bay Water Sample Collection

Seawater used for method testing was sampled from two separate campaigns of opportunity. A ~10 L subsurface seawater sample (at a depth of <1 m) was collected in Delaware Bay on June 14, 2019, onboard the *R/V Joanne Daiber,* operated by the University of Delaware School of Marine Science and Policy. Samples were all collected at a single time point. Further sampling details for this project are included in Burdette et al.[3]

Additionally, subsurface seawater samples (at a depth of ~5 m) were collected from Niskin bottles on a CTD rosette deployed in the North Atlantic Ocean at the Bermuda Atlantic Time Series (BATS) station July 23-31, 2021 (summer), and January 18-26, 2023 (winter), onboard the *R/V Atlantic Explorer*, operated by the Bermuda Institute of Ocean Sciences (BIOS). The Atlantic Ocean seawater samples selected for comparison of the oligotrophic seawater were collected under similar sampling times, locations, and conditions. Oligotrophic seawater has low dissolved organic carbon concentrations.

The Delaware Bay sample was collected in a pre-cleaned 20 L Nalgene HDPE jerrican, and the North Atlantic Ocean seawater was sampled in individual 200 mL pre-cleaned, amber glass bottles. All samples were stored at -20°C and transported to the laboratory for analysis. The true volume of each collected seawater sample was calculated from the measured sample mass and the measured or calculated sample density.

The samples were thawed and filtered with individual, 0.45 µm polyethersulfone membrane syringe filters prior to SPE (unless otherwise specified) to remove particulates. Sample blanks of ultrapure water were collected and treated the same as the seawater.

Text S5. Colorimetric Method Validation

AOT and CTAC solutions had percent recoveries within 10% of 100% recovery. However, SDS and Hyamine solutions had percent recoveries of 120% and 141%, respectively, with measured absorbances outside the 95% prediction interval of their respective calibration curves. This is likely because the concentrations selected were at the upper edge of the calibration curves where there the regression model inherently has less accuracy. Additionally, the anionic and cationic calibration curves were made with a combination of two surfactant standards for each ionic surfactant class, thus representing the broad trend of concentration-dependent absorbances for different ionic surfactants. Individually, surfactants within the same class may complex with the dye and extract into the organic phase, toluene or chloroform, at different efficiencies depending on properties such as alkyl chain length.[4] This could contribute to slight differences in measured absorbance of the dye-complex, resulting in less accurate calculated concentrations from the calibration curves.

For nonionic surfactant Brij, even though the percent recovery is less than 100%, the measurements all fall within the 95% prediction interval of the calibration curve. The lower percent recovery could be due to the chain length variability in the Brij standard and the average molecular weight used to calculate Brij solution concentrations. The nonionic colorimetric method specifies a calibration built on the absorbance response of Brij. Since the complexation of nonionic dye, cobalt thiocyanate, is dependent on the length or number of units in the oxyethylene chain (n_{EO}),[2] the nonionic quantification is more susceptible to variability. The low percent recovery of Genapol, which falls well outside the 95% prediction interval for the nonionic calibration, therefore coincides with its reduced oxyethylene chain length (n_{EO}=10) compared to that of Brij (n_{EO}=23). The Genapol standard solution resulted in a much lower measured absorbance than that of the Brij standard solution. This exhibits the limitations of nonionic surfactant quantification based on Brij as the reference standard, which may not be a holistic representation of nonionic surfactants of the differing oxyethylene chain lengths that may be present in environmental samples. This method targets nonionic surfactants of larger oxyethylene chains, and therefore nonionic surfactants of smaller chain lengths, may not be detected. Additionally, nonionic surfactants without oxyethylene chains will not be quantified with this method.

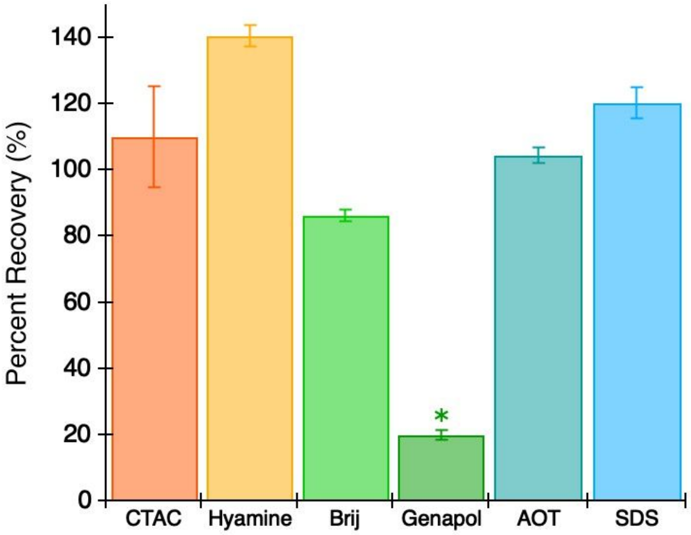

Figure S4. Percent recoveries of individual surfactant solutions quantified with the colorimetric UV-vis methods. Two surfactants for each surfactant class, cationic (oranges) CTAC & Hyamine, nonionic (greens) Brij and Genapol, and anionic (blues) AOT & SDS, were tested. Averages and standard deviation error bars represent three samples. Asterisk denotes sample with two measured sample values below the method detection limit.

Table S2. Average and standard deviations for the percent difference between the true and measured concentration of a surfactant class of interest in the presence of different surfactant classes (interferents) at various concentration levels relative to the target class, and positive percent relative differences represent measured concentrations higher than the actual concentration and negative percent relative differences represent measured concentrations lower than the actual concentration.

**Class of Interest**	**Interferent Level**	**Cationic**	**Nonionic**
**Anionic (SDS)**	Low (1:5)	24.6 ± 6.3 %	19.4 ± 13.4 %
	Equal (1:1)	21.8 ± 14.0 %	32.5 ± 28.4 %
	High (5:1)	4.4 ± 20.7 %	20.0 ± 7.9 %
	**Interferent Level**	**Anionic**	**Nonionic**
**Cationic (CTAC)**	Low	-54.8 ± 7.3 %	-6.8 ± 14.7 %
	Equal	-82.5 ± 2.7 %	27.2 ± 19.7 %
	High	-68.0 ± 3.1 %*	-29.2 ± 16.1 %
	**Interferent Level**	**Anionic**	**Cationic**
**Nonionic (Brij 35)**	Low	-9.7 ± 11.2 %	0.8 ± 3.6 %
	Equal	-13.0 ± 15.0 %	40.1 ± 4.1 %
	High	2.3 ± 14.3 %*+	222.3 ± 13.0 %

*Denotes measured values which fell below limit of detection

+Denotes sample with only two sample replicates

Table S3. The extraction efficiencies of surfactants in 35 g/L NaCl solution measured with the colorimetric method and those measured previously with mass spectrometry.

**Surfactant Class**	**Ext Eff (%)**<br>**6 - 12 mL ACN**	**Ext Eff (%)[a]**<br>**4 mL ACN**
**Anionic** (ENVI-18)	60 – 89%	83 – 99%
**Cationic** (ENVI-Carb)	21 – 46%	75 – 77%
**Nonionic** (ENVI-18)	30 – 42%	63 – 70%

[a]Burdette & Frossard[5]

Table S4. Measured anionic, cationic, and nonionic surfactant concentrations for pure water blank and 0.003 µM glucose solution obtained through the colorimetric UV-vis spectroscopy methods.

**Sample**	**Anionic Conc (µM)**	**Cationic Conc (µM)**	**Nonionic Conc (µM)**
**Pure Water Blank**	0.1492 ± 0.0306	0.0469 ± 0.0015*	0.6006 ± 0.2989*
**0.003 µM Glucose**	0.1739 ± 0.1552	0.0485 ± 0.0029*	0.4590 ± 0.0202*

*Denotes measured values below the limit of detection

Note: Each blank average represents two replicates and each glucose average represents three replicates

Table S5. Different SPE elution compositions tested with separate ENVI-18 and ENVI-Carb extractions of 100 mL unfiltered solutions comprised of NaCl (35 g/L) and a mixture of three surfactants, each from different ionic classes. Target colorimetry sample concentrations for each surfactant was 0.36 µM SDS, 0.36 µM CTAC, and 0.6 µM Brij 35. Resulting extraction efficiencies of each are shown in Figure S5.

**Elution Method**	**Elution Description**
A	8 mL ACN
B	4 mL ACN, 4 mL acetone
C	6 mL ACN, 2 mL ACN/acetone (1:1)
D	4 mL ACN, 4 mL ACN/acetone (1:1)
E	4 mL ACN, 2 mL ACN/acetone (1:1), 2 mL acetone

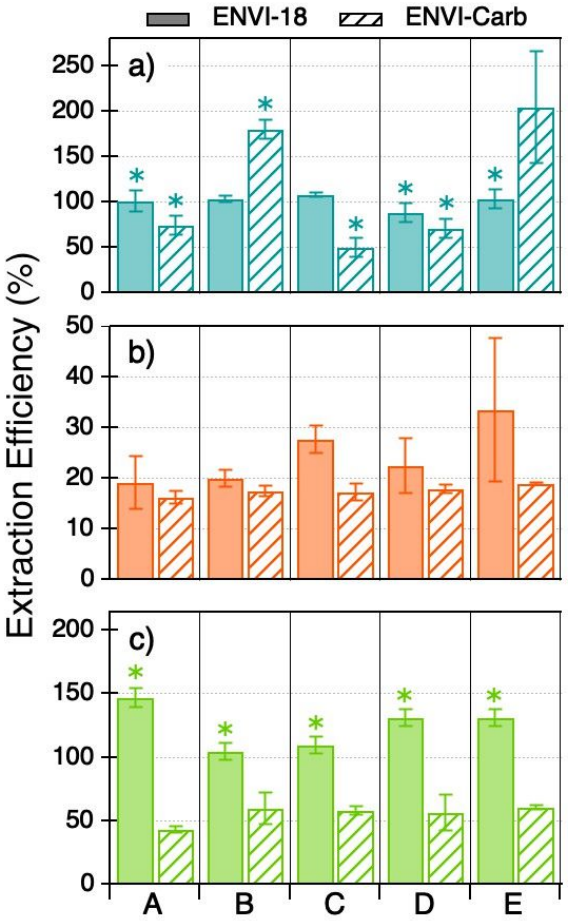

Figure S5. Extraction efficiencies of anionic, cationic, and nonionic surfactants utilizing different 8 mL elution compositions (outlined in Table S5). Error bars represent at least two replicate extractions. Asterisks designate samples that only have one extraction and where the error bars represent the 95% confidence interval for the single sample measurement. Most cationic surfactant concentrations measured were below the detection limit, with only two of the ENVI-18 (one replicate of C and one replicate of E) above detection limit. For nonionic surfactants, all measured values were below the detection limit, due to the low nonionic surfactant concentration in the initial sample. Some measured sample concentrations (and thus extraction efficiencies) of ENVI-18 nonionic surfactants from the same analysis set were very high (upwards of > 300% extraction efficiencies) and therefore were removed, leaving only single sample measurements for those reported here.

References

(1) Nozière, B.; Gérard, V.; Baduel, C.; Ferronato, C. Extraction and characterization of surfactants from atmospheric aerosols. *JoVE (Journal of Visualized Experiments)* **2017**, (122), e55622.
(2) Amirov, R.; Skvortsova, E.; Saprykova, Z. Complexation of cobalt (II) with thiocyanate ions in aqueous solutions of nonionogenic surfactants. *Russian Journal of Coordination Chemistry* **2003**, *29*, 554-558.
(3) Burdette, T. C.; Bramblett, R. L.; Deegan, A. M.; Coffey, N. R.; Wozniak, A. S.; Frossard, A. A. Organic Signatures of Surfactants and Organic Molecules in Surface Microlayer and Subsurface Water of Delaware Bay. *ACS Earth Space Chem.* **2022**, *6* (12), 2929-2943.
(4) Waters, J.; Kupfer, W. The determination of cationic surfactants in the presence of anionic surfactant in biodegradation test liquors. *Anal. Chim. Acta* **1976**, *85* (2), 241-251.
(5) Burdette, T. C.; Frossard, A. A. Characterization of seawater and aerosol particle surfactants using solid phase extraction and mass spectrometry. *J. Environ. Sci.* **2021**, *108*, 164-174.
